# Highly energy-tunable quantum light from moiré-trapped excitons

**DOI:** 10.1126/sciadv.aba8526

**Published:** 2020-09-11

**Authors:** H. Baek, M. Brotons-Gisbert, Z. X. Koong, A. Campbell, M. Rambach, K. Watanabe, T. Taniguchi, B. D. Gerardot

**Affiliations:** 1 Institute of Photonics and Quantum Sciences, SUPA, Heriot-Watt University, Edinburgh EH14 4AS, UK.; 2 National Institute for Materials Science, Tsukuba, Japan.

## Abstract

Photon antibunching, a hallmark of quantum light, has been observed in the correlations of light from isolated atomic and atomic-like solid-state systems. Two-dimensional semiconductor heterostructures offer a unique method to create a quantum light source: Moiré trapping potentials for excitons are predicted to create arrays of quantum emitters. While signatures of moiré-trapped excitons have been observed, their quantum nature has yet to be confirmed. Here, we report photon antibunching from single moiré-trapped interlayer excitons in a heterobilayer. Via magneto-optical spectroscopy, we demonstrate that the discrete anharmonic spectra arise from bound band-edge electron-hole pairs trapped in moiré potentials. Last, we exploit the large permanent dipole of interlayer excitons to achieve large direct current (DC) Stark tuning up to 40 meV. Our results confirm the quantum nature of moiré-confined excitons and open opportunities to investigate their inhomogeneity and interactions between the emitters or energetically tune single emitters into resonance with cavity modes.

## Teaser

Photon antibunching, a hallmark of quantum light, is observed from moiré-trapped interlayer excitons in a 2D heterobilayer.

## INTRODUCTION

The ability to stack unlimited combinations of atomic layers with arbitrary crystal angle (θ) has opened an innovative paradigm in quantum material design. For example, easily tunable Bloch minibands emergent in moiré lateral superlattices have enabled remarkable observations with graphene heterostructures, such as nearly flat bands with narrow bandwidths at specific θ (*1*) that can lead to superconductivity (*2*) and correlated insulator states (*3*). Beyond graphene, unique opportunities arise with transition metal dichalcogenide (TMD) semiconductors, where band-edge electrons and holes located at two degenerate, but inequivalent, corners of the Brillouin zone (±**K** valleys) form excitons with a strong Coulomb interaction (*4*). Because of strong spin-orbit coupling, the carriers exhibit locked spin and valley degrees of freedom. The in-plane 2π/3 rotational (Ĉ_3_) symmetry of the monolayer (ML) TMD crystal structure generates valley contrasting optical selection rules for strongly bound excitons (*5*). Stacking any two different ML TMDs creates a heterobilayer with type II band alignment (*6*), which exhibits spatially indirect interlayer excitons (IXs) with highly tunable photoluminescence (PL) energy (*7*–*10*). The twist of a TMD heterobilayer changes the displacement of the constituent ±**K** valleys, determining the coupling of IXs to the light cone (*11*): For samples with θ ~ 0° or 60°, bright IX emission is observed (*7*–*10*). Conversely, for samples with large deviations from θ ~ 0° or 60°, minimal PL intensity is observed from the IX due to a large momentum shift between the band-edge electrons and holes (*12*–*14*). Similar to graphene bilayers (BLs), the constituent TMD MLs also interact with each other and create moiré potentials dependent on θ, which can hybridize wave functions across both layers (*14*–*16*) or lead to uniform high-density arrays of quantum emitters (*17*, *18*) or topological bands whose properties can be manipulated by electric or strain fields (*19*–*21*).

Absorption and PL of TMD heterobilayer samples have recently been investigated to probe for moiré trapping potentials (*22*–*26*). In the limit of low temperature and weak excitation, PL spectra exhibit sharp lines, similar to III-V or WSe_2_ quantum dots (*27*–*29*) except for strong helical polarization due to Ĉ_3_ symmetry of the constituent crystal lattices and a notable absence of observable fine structure (*22*, *23*). In addition, highly uniform *g*-factors dependent on relative layer twist are observed, clear fingerprints of the spin and valley configurations for excitons composed of band-edge electrons and holes at the ±**K** points. Last, the helical polarization appears to be determined by the atomic registry. Combined, these observations provide compelling evidence for moiré-trapped IXs. Nevertheless, ambiguity remains about their precise nature. Do the sharp spectral features arise from single trapped excitons? Why are the spectral features inhomogeneous, unlike the *g*-factors? Here, we provide unambiguous proof of the quantum nature of the moiré-trapped IXs via the observation of photon antibunching. This opens a route to investigate second-order cross-correlations among the distinct spectral peaks and to understand the full nature of moiré quantum emitter arrays. Furthermore, by incorporating the moiré-trapped IXs into a device that enables an applied out-of-plane electric field, we achieve 40-meV tuning of the quantum emitters emission energy via the DC Stark effect. This enables a precise measurement of the large permanent dipole of the quantum emitters, which results from the electron-hole pair separation in the heterobilayer. Ultimately, our results may lead to engineering highly tunable arrays of coherent quantum emitters and spin-photon interfaces.

## RESULTS

### MoSe_2_/WSe_2_ moiré heterobilayer samples

**Fig. 1:**
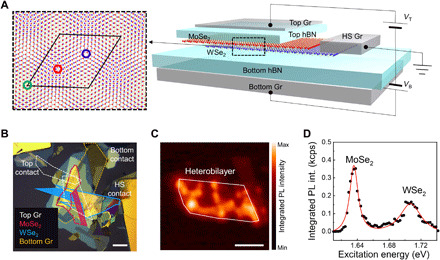
MoSe_2_/WSe_2_ moiré heterobilayer. (**A**) Schematic of sample 1, the moiré heterobilayer with dual gates. Graphite (Gr) layers are used as contacts for top, bottom, and heterostructure (HS), and hBN layers are used for both dielectric layers and encapsulation of heterobilayer. Illustration of a moiré superlattice with a twist angle of ~60° is displayed in the dashed box. The moiré supercell is represented as a black solid line, and the three circles (in red, blue, and green) indicate the high-symmetry points under Ĉ_3_ operation. (**B**) Optical micrograph of sample 1. The top and bottom graphite layers are represented as white and orange dashed lines, respectively, and the MoSe_2_ and WSe_2_ flakes are outlined in magenta and blue solid lines, respectively. The filled regions represent ML TMDs. (**C**) Low-temperature spatial PL intensity map of the IXs (energy range of 1.38 to 1.40 eV). The heterobilayer is outlined with a white solid line. (**D**) Low-temperature PLE intensity (int.) plot of a representative IX in the heterostructure, showing two resonances corresponding to the intralayer exciton states in ML MoSe_2_ and ML WSe_2_. Intensity scale is in kilo counts per second (kcps). Scale bars, 20 μm (B) and 5 μm (C).

Our heterobilayer samples consist of ML MoSe_2_ and ML WSe_2_ encapsulated by hexagonal boron nitride (hBN). A MoSe_2_/WSe_2_ heterobilayer stacked with a small twist angle forms a periodic moiré superlattice as presented in Fig. 1A. To apply vertical electric field to the moiré heterobilayer, we first investigate a dual gated device (sample 1) fabricated using graphite and hBN as electrical contact and dielectric layers, respectively, as shown in Fig. 1A (see section S2). Figure 1B shows an optical micrograph of the fabricated device. As determined by the cleaved edges of MoSe_2_ and WSe_2_, the bright IX intensity, and Landé *g*-factors described below, the twist angles for the heterobilayer samples that we investigate are close to 60° (*30*). Graphite layers, which form the top and bottom gates of the heterobilayer, were connected to prepatterned Au electrodes. The thicknesses of the top and bottom hBN are determined via high-resolution ellipsometry to be 17.4 ± 0.2 nm and 18.2 ± 0.3 nm, respectively. A second moiré heterostructure sample (sample 2) is engineered for enhanced collection efficiency. Here, we place the heterostructure on a gold mirror and choose the hBN bottom layer thickness (96 nm) to position the heterobilayer at an antinode of the electric field to create a planar antenna (see section S3) (*31*). This helps improve the signal of the spatially indirect IXs, which has an intrinsically small oscillator strength (*7*).

A low-temperature (4 K) confocal PL intensity map from sample 1 in a photon energy range of 1.38 to 1.40 eV is shown in Fig. 1C. IX emission across wide regions of the heterobilayer is detected, indicating a clean interface between the MoSe_2_ and WSe_2_ layers. While the spectral positions and intensity of the moiré-trapped IX vary across the heterobilayer region at low excitation powers, the *g*-factors and polarization dependence across the sample indicate a uniform atomic registry for the moiré-trapped IX. In addition to the heterobilayer, a BL MoSe_2_ region can also be identified in the top right region of the map by its indirect emission. To confirm that the origin of the heterobilayer PL signal arises from IXs, we perform PL excitation (PLE) spectroscopy, in which a continuous wave (CW) excitation laser is scanned from 1.61 to 1.75 eV while monitoring the intensity of IX PL at ~1.4 eV. Figure 1D, the PLE spectrum, features two prominent resonances at ~1.63 and ~1.70 eV, which correspond to the absorption of the intralayer 1s state of A excitons in ML MoSe_2_ and WSe_2_, respectively. To exploit the high quantum yield at resonant excitation of MoSe_2_, most of the PL spectroscopy results reported here are obtained with an excitation energy of 1.63 eV. Similar results are obtained from sample 2.

### Magneto-PL spectroscopy

**Fig. 2:**
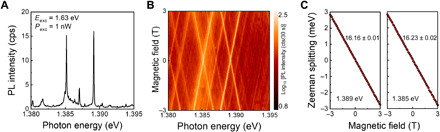
Magneto-PL spectroscopy of moiré-trapped excitons. (**A**) Representative PL spectra of moiré IXs with an excitation energy of 1.63 eV at 1 nW power. (**B**) Magnetic field dependence of the moiré IXs in the *2H*-type MoSe_2_/WSe_2_ heterostructure as a function of the applied out-of-plane magnetic field. (**C**) Plots of Zeeman splitting energy versus magnetic field with linear fits for the peaks at 1.389 and 1.385 eV. Intensity scale is in counts (cts) per 30 seconds.

Figure 2A shows a representative low-temperature confocal PL spectrum of moiré IXs. The PL spectrum reveals several discrete spectra with emission energies at ~1.39 eV, in agreement with recently reported values (*22*, *23*). Peak linewidths of ~100 μeV are observed at low excitation powers; the peak at 1.389 eV has a linewidth of 80 μeV. Such linewidths are similar to previously reported values in moiré-trapped IXs and two orders of magnitude smaller than the ones from broad IXs (typically 7 to 30 meV) (*8*–*10*, *32*, *33*). To confirm that the discrete peaks arise from band-edge states at the ±**K** points, we perform magneto-optical spectroscopy in Faraday configuration. A clear linear Zeeman splitting for each IX peak is observed, as shown in Fig. 2 (B and C), and an average *g*-factor of −16.2 is extracted, in strong agreement with the gyromagnetic ratios reported for IX ensemble emission (*34*) and moiré-trapped IXs with 2*H* (θ ~ 60°) stacking (*22*, *23*). The linear Zeeman splitting persists to very small applied magnetic fields, indicating a lack of fine-structure splitting, which can arise due to an asymmetric confinement potential. In addition, polarization-resolved PL reveals that the moiré-trapped IX exhibits circular polarization, which is copolarized to the excitation polarization for excitation resonant with the 1s intralayer resonance of WSe_2_ (see figs. S5 and S6). The combination of *g*-factor values and copolarized emission implies that moiré excitons are confined in the same atomic configuration, likely $H_{h}^{h}$ (*17*, *23*), as indicated by the blue circle in Fig. 1A. Here, *H* corresponds to θ ~ 60° while the superscript and subscript represent the hexagon center of the electron and hole layers, respectively. Together, these results demonstrate that the IXs are composed of bound band-edge electron-hole pairs at the ±**K** points trapped in rotationally symmetric moiré potentials.

### DC Stark tuning

**Fig. 3:**
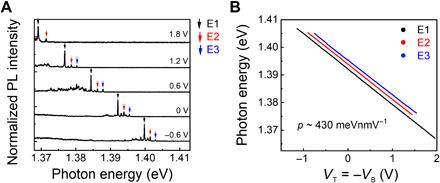
Stark tuning of moiré-trapped excitons. (**A**) PL spectra of IXs at different gate voltages. Three representative peaks are indicated as E1, E2, and E3. (**B**) Plot of emission energy versus gate voltage for E1, E2, and E3 peaks. Values of electrical dipole moments of ~420 meVnmV^−1^ are determined by the linear fits.

Using the top and bottom gates, the dependence of moiré IXs on external electric field is investigated as shown in Fig. 3 (A and B). Since the thicknesses of the top and bottom hBN are very similar, gate voltages for top (*V*_T_) and bottom (*V*_B_) are set to the same magnitude but opposite direction to apply a vertical electric field without strongly affecting the Fermi energy of the heterobilayer. The peak positions of all of moiré IXs linearly shift with applied electric field. Three representative peaks, indicated as E1, E2, and E3, are highlighted in Fig. 3A. A tuning range of ~40 meV is observed, much larger than that of ML WSe_2_ quantum dots, which have minimal out-of-plane permanent dipole (*29*, *35*). Figure 3B shows a plot of photon energy versus gate voltage for E1, E2, and E3 peaks with a step size of 0.01 V. Using the equation Δ*U = −pE* (where Δ*U* is the linear Stark shift, *p* is the out-of-plane electric dipole moment, and *E* is the vertical electric field) with $E=\frac{\left(V_{T}-V_{B}\right)}{t_{\text{hBN}}}\frac{\varepsilon_{\text{TMD}}}{\varepsilon_{\text{hBN}}}$ [where ε_TMD_ = 7.2 and ε_hBN_ = 3.8 are the relative permittivity of TMD and hBN, respectively (*36*, *37*), and *t*_hBM_ is the total thickness of top and bottom hBN], the average electrical dipole moment is calculated as 429 ± 4 meVnmV^−1^. Since the magnitude of dipole moment is expressed as *p = ed*, where *e* is the single electron charge and *d* is the electron-hole separation, *d* is estimated as 0.429 ± 0.004 nm.

### Photon antibunching

**Fig. 4:**
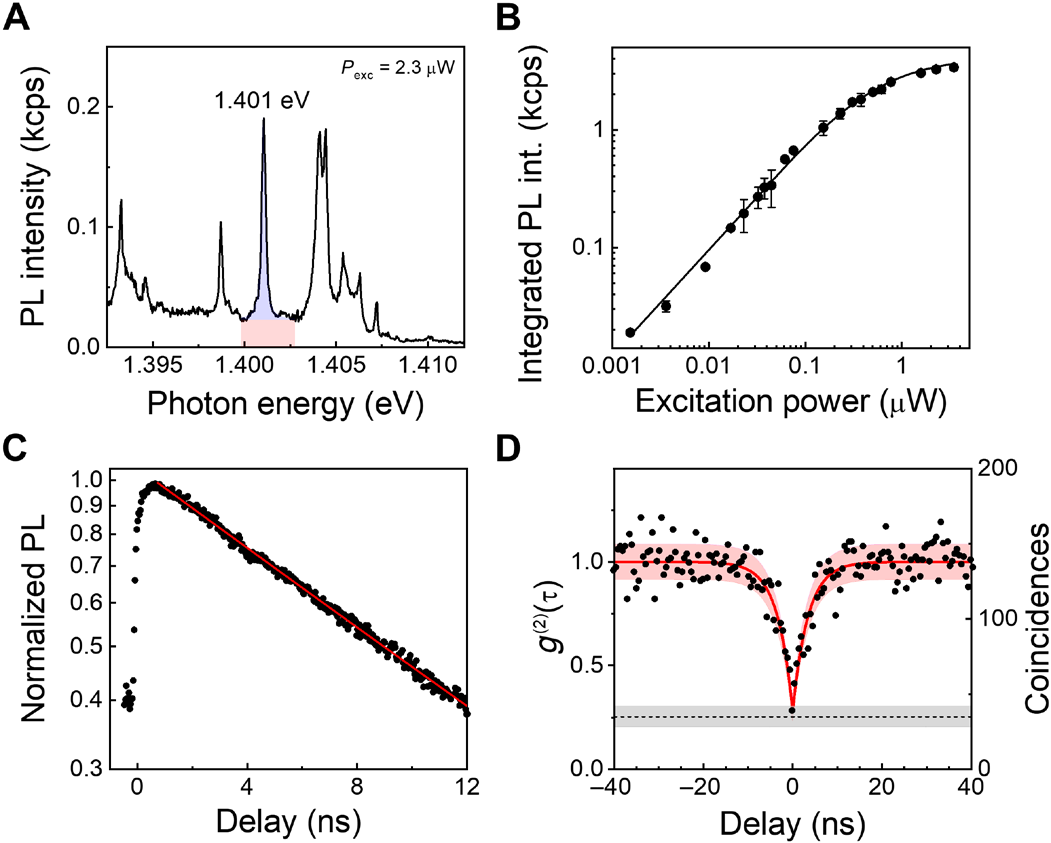
Quantum nature of moiré IXs. (**A**) PL spectrum from sample 2. The emitter at 1.401 eV is used for power-dependence, TRPL, and second-order photon correlation measurement. The blue and red regions represent the estimated PL signal from the emitter and the background. (**B**) Integrated PL intensity of a single emitter at different excitation powers. (**C**) Time-resolved normalized PL intensity of the single emitter under 80-MHz pulsed excitation at 1.63 eV with an average excitation power of 4 μW. The red solid line shows a single exponential decay fit to the experimental data, revealing a lifetime of 12.1 ± 0.3 ns. (**D**) Second-order photon correlation statistics using 2.3 μW CW excitation at 760 nm (black dots) show clear antibunching. The red solid line represents a fit of the experimental data, revealing a *g*^(2)^(0) = 0.28 ± 0.03. The red shadowed area represents the Poissonian interval error associated to the experimental determination of *g*^(2)^(τ). The black dashed line represents the experimental limitation for *g*^(2)^(0) owing to the nonfiltered emission background. The gray shadowed area shows the error interval in the determination of the limitation for *g*^(2)^(0).

To characterize the quantum nature of moiré IXs, the power-dependent PL and second-order correlation function *g*^(2)^(τ) are measured for a single emitter. These measurements are performed on sample 2, which is engineered for enhanced collection efficiency. We identify that sample 2’s MoSe_2_/WSe_2_ stacking orientation is ~60° by the IX spin-valley fingerprints revealed in the magneto-optics (see fig. S7). Figure 4A shows a PL spectrum that includes the emitter at 1.401 eV, which we target for the photon antibunching experiment, chosen because of its relative brightness and minimal background. The emission intensity of the moiré IX saturates with increasing excitation power, as shown in Fig. 4B, hinting at the confined nature. The fit is based on $I=I_{\text{sat}}(\frac{P_{\text{exc}}}{P_{\text{exc}}+P_{N}})$, where *I* is the PL intensity, *I*_sat_ is the saturation intensity, *P*_exc_ is the excitation power, and *P_N_* (0.47 μW) is the excitation power at which *I* = *I*_sat_/2. The emission peak is spectrally filtered for time-resolved PL (TRPL) and *g*^(2)^(τ) measurements. Figure 4C shows the TRPL intensity trace of the emitter. The solid red line represents a fit with a single exponential decay function with a decay time of *T*_1_ = 12.1 ± 0.3 ns, within the range reported for IX ensemble emission in MoSe_2_/WSe_2_ heterobilayers (2 to 100 ns) (*7*, *34*, *38*). Next, we send the filtered spectrum to a Hanbury Brown–Twiss interferometer. Figure 4D shows the second-order photon correlation statistics. The red solid line represents a fit using *g*^(2)^(τ) = 1 − ρ^2^*e*^−∣τ∣/τ^*_c_*, where τ is the time delay between two consecutive detected photons, τ*_c_* is the decay time, and ρ *=* SBR/(SBR + 1), with SBR being the signal-to-background ratio. The fit reveals *g*^(2)^(0) = 0.28 ± 0.03 and τ*_c_* = 4.3 ± 0.2 ns. The decay time τ*_c_* is inversely proportional to the lifetime (*T*_1_) and the CW pump rate *W_p_*: τ_c_ = 1/(*T*_1_ *+ W_P_*) (*39*). Based on this, our fits to the *g*^(2)^(τ) data in Fig. 4D indicate an excitation pump rate *W_p_* ~ 2*T*_1_. The orange shadowed area represents the Poissonian interval error associated with the experimental determination of *g*^(2)^(τ). The *g*^(2)^(0) value is well below the threshold of 0.5, unambiguously proving the quantum nature of the light emitted by the moiré-trapped IXs. Last, the black dashed line and the gray shadowed area represent the average and error interval of the experimental limitation for *g*^(2)^(0), respectively, owing to the nonfiltered emission background (section S8), which results in an average SBR of 6.4. The results suggest that spectrally isolated moiré-trapped IXs offer potential as high-purity single-photon sources and coherent spin-photon interfaces.

## DISCUSSION

Via the observation of photon antibunching, we demonstrate the quantum nature of discrete anharmonic spectra from moiré-confined excitons in a two-dimensional (2D) heterostructure. The quantum emitters observed from the MoSe_2_/WSe_2_ heterobilayers are identified to originate from confined IXs by a moiré potential as confirmed by magneto-optical spectroscopy. The uniform *g*-factor, lack of fine-structure splitting, and helical polarization are distinguishing features of the moiré potential, which preserves the intrinsic Ĉ_3_ symmetry of the constituent crystal lattices. In contrast, localized excitons in ML TMDs generated by extrinsic defects or strain exhibit a large fine-structure splitting, large variations in *g*-factor, and linearly polarized emission (*28*, *29*).

In addition, we establish that the emission energy of moiré IXs can be highly and reliably tuned using the DC Stark effect: We achieve 40-meV tuning in total, substantially larger than reports for other solid-state emitters (*40*). Since electrons and holes reside in spatially separated layers, the out-of-plane electrical dipole moment of IXs is much larger than that of intralayer excitons. The electron-hole separation, *d*, is estimated as 0.429 ± 0.004 nm, in good agreement with previously estimated values (0.5 to 0.6 nm) of broad IXs (*9*, *10*). The slightly smaller value for *d* obtained here might be due to the interaction between the two layers, which causes the moiré potential and provides the carrier localization. Moiré IXs are confined in a specific atomic registry, and it is expected that the interlayer distances at trapping sites are 0.6 to 2 Å (*17*, *41*) closer than other locations. Thus, moiré-trapped IXs can have slightly reduced values of electric dipole moment compared to broad (nontrapped) IXs, where the modulation of interlayer distance might be averaged out. The broad spectral tunability of moiré IXs combined with the single photon nature enables the pursuit of promising technological applications. The energy tuning can be used to bring moiré quantum emitters into resonance with a cavity for Purcell enhancement for the generation of indistinguishable photons.

From a fundamental point of view, the unambiguous demonstration of quantum light emission from moiré-confined IXs is a crucial building block to better understand the underlying physics of excitons in moiré superlattice potentials. Second-order cross-correlation measurements between neighboring spectral peaks can be used to understand their relationship, addressing questions regarding the perceived inhomogeneity in the moiré superlattice and the possibility of strong interactions within the excitonic superlattice, which can lead to superradiance, topological moiré minibands, or the realization of a tunable Mott–Hubbard Hamiltonian (*19*–*21*, *42*–*46*).

## MATERIALS AND METHODS

### Fabrication of moiré heterostructures

ML MoSe_2_ and WSe_2_ layers (from 2D semiconductors) were first prepared separately on polydimethylsiloxane (PDMS), and then, one layer was transferred onto the other, resulting in a MoSe_2_/WSe_2_ heterostructure on PDMS. In this method, the heterobilayer interface remains pristine, without exposure to polymer. Meanwhile, graphite and hBN layers were also prepared on PDMS. These prepared 2D materials were stacked successively onto Au electrode–patterned SiO_2_/Si substrates using a dry transfer technique in an Ar-filled glove box.

### PL spectroscopy

A confocal microscope with an objective lens with a numerical aperture of 0.82 was used for μ-PL measurements with excitation λ = 759.6 and 727.0 nm for resonant excitation of MoSe_2_ and WSe_2_ A excitons, respectively. The sample was placed on piezoelectric stage for nano-positioning at 4 K in a closed-cycle cryostat with a superconducting magnet. The PL signal was dispersed in a 500-mm focal length spectrometer and detected by nitrogen-cooled charge-coupled device with a spectral resolution of ∼70 μeV at λ = 900 nm for 1200 lines/mm. Polarization-dependent PL was measured by changing the relative angle between a quarter–wave plate and a linear polarizer. A fiber-based Hanbury Brown and Twiss interferometer was used for second-order correlation measurements after spectral filtering (resolution, 0.15 nm) of the PL signal. Photons were counted using superconducting nanowire single-photon detectors.

## Supplementary Material

aba8526_SM.pdf

## SUPPLEMENTARY MATERIALS

Supplementary material for this article is available at http://advances.sciencemag.org/cgi/content/full/6/37/eaba8526/DC1
