# Supplementary material for: Highly energy-tunable quantum light from moiré-trapped excitons
Source: Sci Adv. 2020 Sep 11;6(37):eaba8526. doi: 10.1126/sciadv.aba8526 (PMC7486092; doi:10.1126/sciadv.aba8526)
Supplement: aba8526_SM.pdf [file aba8526_SM.pdf]

Science Advances
AAAS

advances.sciencemag.org/cgi/content/full/6/37/eaba8526/DC1

Supplementary Materials for

Highly energy-tunable quantum light from moiré-trapped excitons

H. Baek*, M. Brotons-Gisbert, Z. X. Koong, A. Campbell, M. Rambach, K. Watanabe, T. Taniguchi, B. D. Gerardot*

*Corresponding author. Email: h.baek@hw.ac.uk (H.B.); b.d.gerardot@hw.ac.uk (B.D.G.)

Published 11 September 2020, *Sci. Adv.* **6**, eaba8526 (2020)
DOI: 10.1126/sciadv.aba8526

This PDF file includes:

Sections S1 to S6
Figs. S1 to S8

Section S1. Optical microscope images of flakes

Figure S1 shows optical microscope images of WSe_2, $MoSe_2$, and heterostructure layers on PDMS. Zigzag edges are indicated as black lines for WSe_2 and $MoSe_2$ flakes. These edges are aligned to fabricate a heterobilayer with a twist angle close to 0 or 60°. From the optical image of the heterostructure, the angle between these edges is determined to be 56.4±1°.

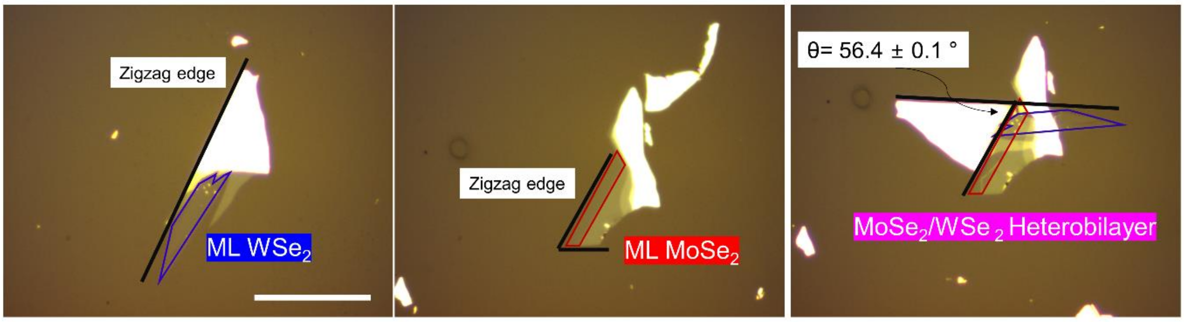

Fig. S1. Optical microscope images of flakes before and after stacking. WSe_2 and $MoSe_2$ monolayers are outlined in blue and red lines, respectively. The scale bar is 50 μm.

Section S2. Gate-tunable $MoSe_2/WSe_2$ heterostructure fabrication

Figure S2 shows optical microscope images of Sample 1. Outlines indicate layers transferred in each step.

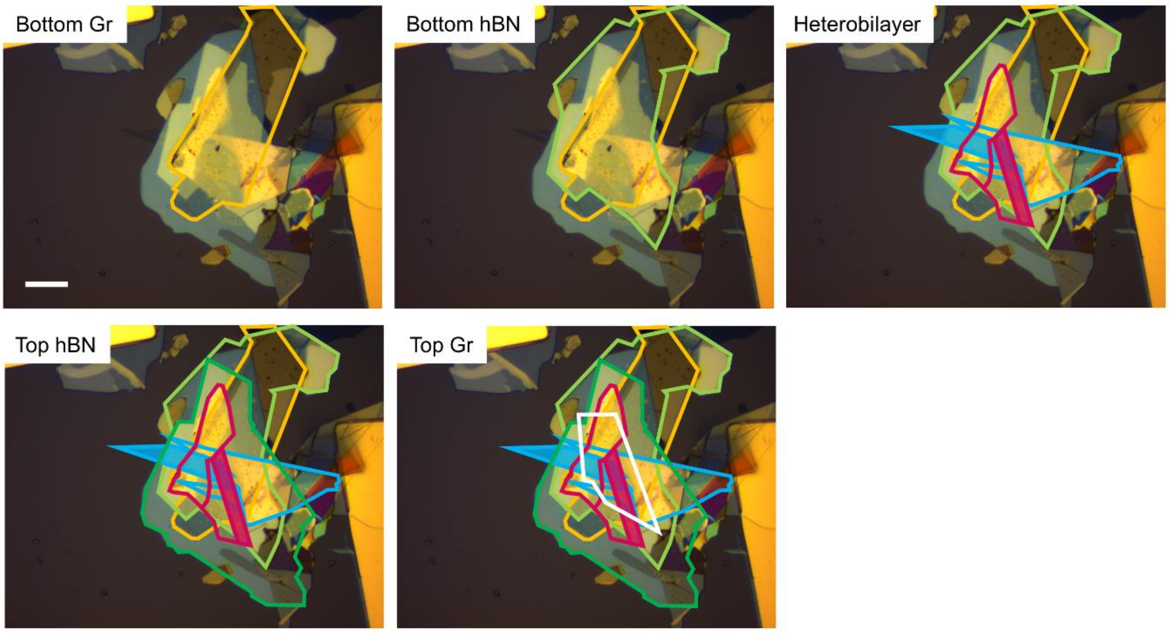

Fig. S2. Optical microscope images of $MoSe_2/WSe_2$ heterostructure. Layers in each transfer step are outlined with different colors. The scale bar is 20 μm.

Section S3. Enhancing the emission intensity of moiré trapped IXs

As discussed in the main manuscript, the interlayer nature of the moiré-trapped excitons in $MoSe_2/WSe_2$ heterostructures gives rise to a small oscillator strength for these quantum emitters. Such an intrinsically small oscillator strength results in a low PL signal intensity, which has hampered the demonstration of the quantum nature of these excitons to date.

With the aim of enhancing the intensity of the PL from the moiré trapped excitons and enabling the second-order correlation ($g^{(2)}$) measurements shown in Fig. 4D, a $MoSe_2/WSe_2$ heterostructure (Sample 2) was fabricated and deposited on top of an a gold mirror with an optimized bottom hBN thickness. The thickness of the bottom hBN was engineered to enhance both the far-field PL intensity and the PL collection efficiency in our fiber-coupled confocal microscope. The far-field PL of the moiré IXs was simulated by employing the analytical model based on the combination of a transfer-matrix method and a dipole emission source term, which allows to simulate the PL emission of both single emitters and intralayer excitons of different 2D materials *(31)*. Figure S3 shows a sketch of the planar multilayer structure used in our simulations. The PL emission from the moiré trapped IXs was simulated by an in-plane oscillating dipole, in agreement with recent theoretical calculations *(11, 18)*. The emitting dipole was placed on top of the hBN flake at a distance of 0.7 nm from the hBN/air interface. The hBN flake was placed on top of a semi-infinite planar gold substrate. In the simulations, we included the refractive indices of hBN and Au reported in references *Phys. Status Solidi B, 256, 1800417 (2019)* and *Opt. Express 25, 25574-25587 (2017)*, respectively.

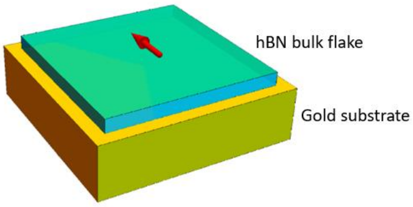

Fig. S3. Sketch of the planar multilayer structure used in our simulations. The red arrow represents the in-plane oscillating dipole employed to simulate the PL emission of the localized IXs.

In our simulations, we calculated the far-field PL of moiré trapped excitons emitting at a wavelength of 900 nm as a function of the hBN thickness. The far-field PL collected by a fiber-coupled confocal microscope (P_{col}) can be expressed as

$$P_{col} \propto \eta_{fibre}\eta_{NA}F_P P_0,$$

with P_0 being the total PL intensity radiated by the corresponding dipole in free space, and F_P being the Purcell enhancement of the spontaneous emission induced by the planar structure. η_{NA} represents the objective collection efficiency, i.e., the ratio of emitted PL that can be collected by an objective with a given numerical aperture (NA). In our simulations, we considered an objective with a NA = 0.82 to match our experimental conditions. η_{fibre} represents the fiber coupling efficiency of our fiber-based confocal microscope. η_{fibre} can be quantified by the overlap integral

$$\eta_{fibre} = \frac{|\int E_{PL}^{*}E_{SMF}dA|^2}{\int|E_{PL}|^2 dA \int|E_{SMF}|^2 dA},$$

where E_{PL} and E_{SMF} are the complex electric fields of the emitted PL signal and the fundamental mode of a single-mode fiber (780HP), respectively, in the same plane (i.e. in a

plane between the microscope objective and the lens used to couple the PL into the single-mode fiber).

Figure S4(a) shows the calculated far-field PL intensity collected by our fiber-coupled confocal microscope normalized by the total radiated power (P_{col}/P_0) as a function of the hBN thickness. As can be seen this figure, two clear maxima with $P_{col}/P_0 > 70\%$ can be observed for hBN thicknesses ranging between 75-100 nm and 295-320 nm. For the fabrication of Sample 2, we employed a hBN bulk flake with a measured thickness of 96 nm.

Finally, Figs. S4(b-d) show the corresponding dependence of the F_P, η_{NA} and η_{fibre}, respectively, as a function of the hBN thickness. As can be seen in these figures, although the collection efficiency of our objective is not very large (~40%), the strong Purcell factor induced by our simple planar structure (~1.7) allows us to collect ~70% of P_0 into our single-mode fiber.

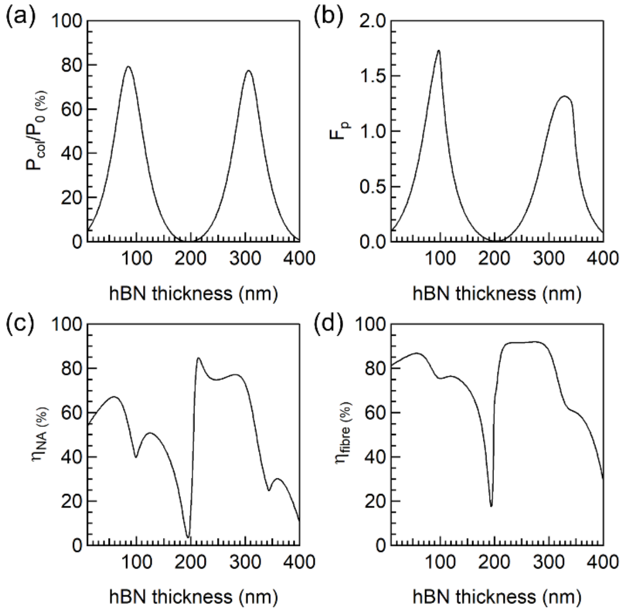

Fig. S4. Simulation results as a function of hBN thickness. Calculated evolution of P_{col}/P_0 (a), F_P (b), η_{NA} (c), and η_{fibre} (d) as a function of the hBN thickness.

Section S4. Polarization-resolved PL spectroscopy

Figure S5 shows circular polarization-resolved PL spectra of moiré IXs. For excitation with circular polarization, the excitation energy is set to 1.75 eV for clearer degree of circular polarization. The peaks show co-polarized characteristics.

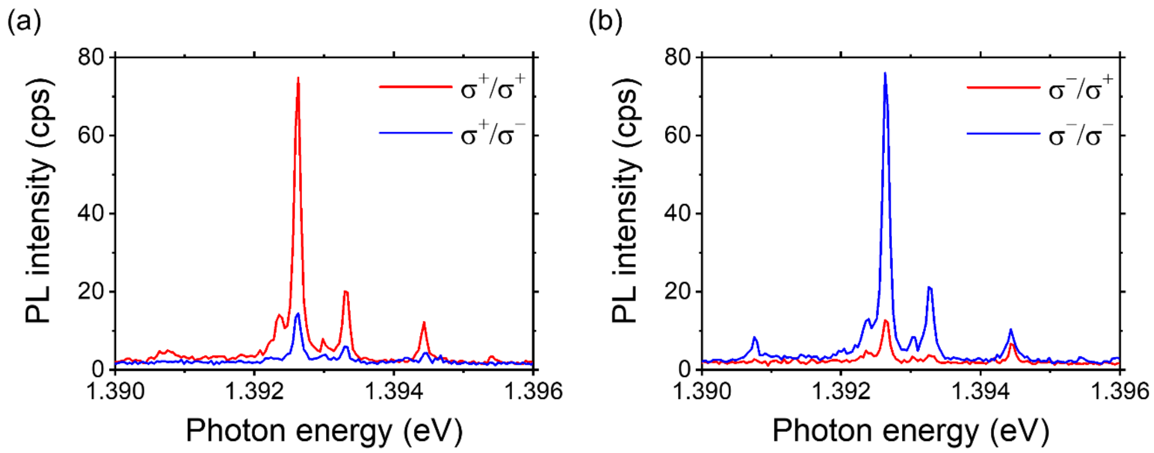

Fig. S5. Circular polarization of moiré IXs. PL spectra of moiré IXs with circular polarization of σ^+ (red) and σ^- (blue) under σ^+ (a) and σ^- excitation (b).

Figure S6 shows σ^+ and σ^- PL spectra as a function of magnetic field. All of peaks shift in the same direction as the magnetic field changes, implying moiré excitons are confined in the same atomic configuration, likely H_h^h (*17*, *23*), indicated as blue circle in Fig. 1A.

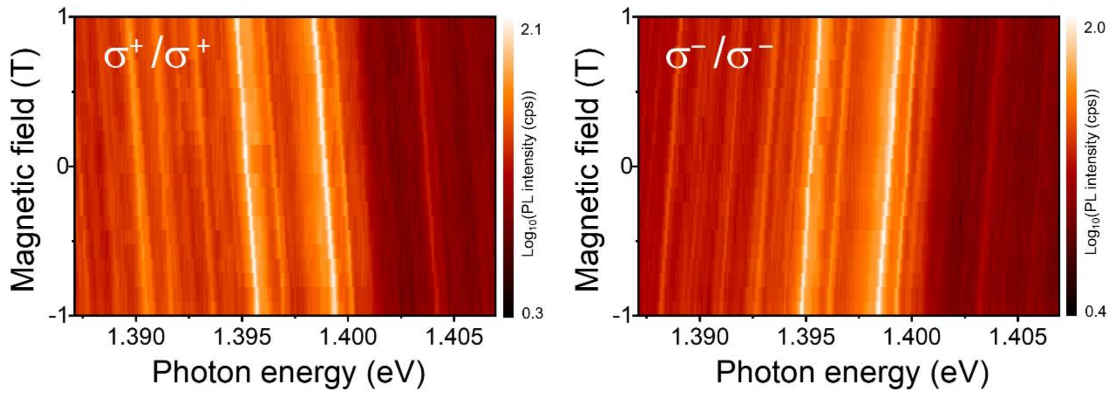

Fig. S6. Magnetic field dependence of polarization-resolved PL emission. PL spectra (σ^+ and σ^-) measured at different magnetic field from -1 to 1 T.

Section S5. Magneto-PL spectroscopy in a second moiré heterostructure sample

Figure S7(a) shows magnetic field dependence of PL emission in a second heterostructure sample. The peak at 1.402 eV is the emission used for photon correlation measurement in Fig. 4. An applied bias was used to maximize the ratio of PL intensity to background for the photon correlation measurement, shifting the peak position in Fig. 4A by about 1 meV compared to the spectrum shown in Fig. S7(a). All emission lines display a linear Zeeman splitting with uniform g-factors. As shown in Fig. S7(b), g-factors for peaks at 1.405 and 1.402 eV are very similar, and these values are in a good accordance with previously reported ones for 2*H* stacked heterostructures.

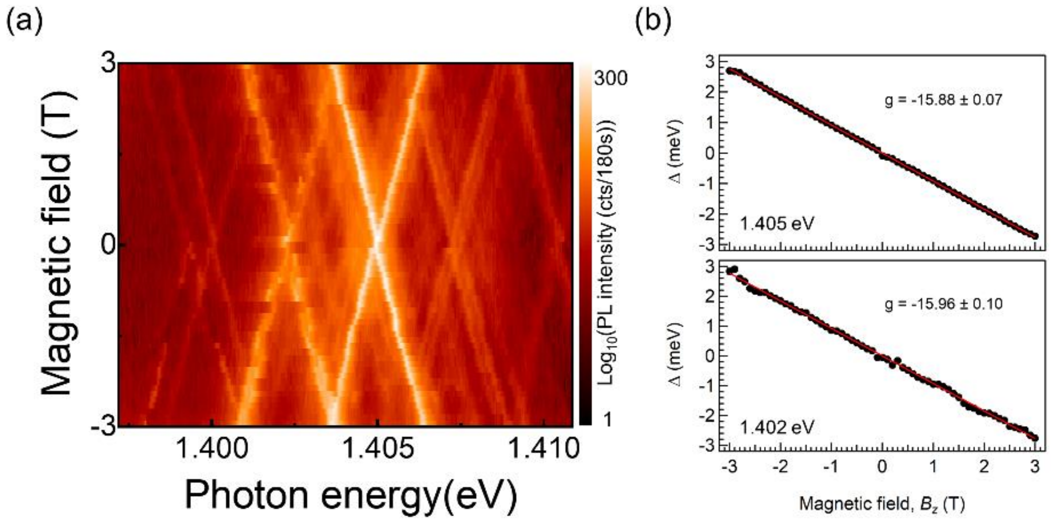

Fig. S7. Magnetic field dependence of PL emission in Sample 2. (a) Magnetic field dependence of the moiré IXs as a function of the applied out-of-plane magnetic field. (b) Plots of Zeeman splitting energy versus magnetic field with linear fits for the peaks at 1.405 and 1.402 eV.

Section S6. Experimental limitation of second-order correlation at zero delay

As discussed in the main manuscript, the experimental value for the normalized second-order photon correlation at zero delay $g^{(2)}(0)$ shown in Fig. 4D is limited by non-filtered emission background. Figure S8(a) shows a high-spectral-resolution PL spectrum of the moiré quantum emitter at 1.401 eV acquired with the same experimental conditions employed for the second-order photon correlation measurements shown in Fig. 4D of the main manuscript prior to spectral filtering. The red and blue shadowed regions represent the estimated background and PL emission of the emitter as obtained from a Lorentzian fit of the PL peak. After spectral filtering with a band-pass filter centered at the emission energy of the emitter (see top panel of Fig. S8(b)). The *SBR* of 6.38 is estimated (see bottom panel of Fig. S8(b)), which limits the experimental determination of $g^{(2)}(0)$ to a minimum a value of $g^{(2)}(0) = 0.25$. In order to estimate the experimental error in the determination of the limitation for $g^{(2)}(0)$, we measured the temporal evolution of the PL of the over 5 minutes (see Fig. S8(c)). As can be seen in this figure, the emission energy of the emitter presents spectral wandering, which results in a time-dependent *SBR* after spectral filtering of the PL spectrum as a consequence of the detuning of the PL signal from the central energy of the filter. Figure S8(d) shows the temporal evolution of the integrated PL intensity of the emitter through the spectral filter over 5 minutes. Unfortunately, this emitter, which was most optimal in terms of signal to noise, exhibited unusually large spectral fluctuations which led to intensity fluctuations after the filter. The dashed line and the yellow shadowed area represent the average value and the standard deviation of the integrated PL intensity, respectively, from which we estimate an experimental error ~0.06 in the determination of the limitation for$g^{(2)}(0)$.

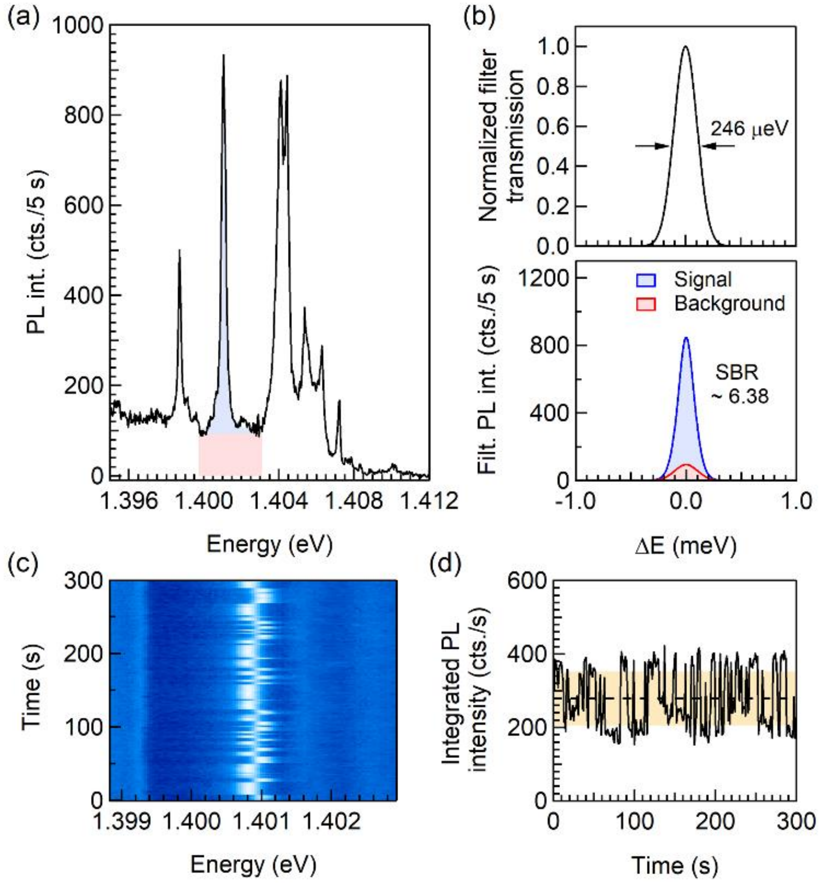

Fig. S8. Limitation in the determination of $g^{(2)}(0)$. (a) High-spectral-resolution PL spectrum (prior to spectral filtering) of the moiré quantum emitter acquired with the same experimental conditions employed for the second-order photon correlation measurements shown in Fig. 4D of the main manuscript. The red and blue shadowed regions represent the estimated background and PL emission of the emitter as obtained from a Lorentzian fit of the PL peak. (b) Normalized experimental transmission of the band-pass filter used to spectrally isolate the PL signal of the emitter at 1.401 eV. The bottom panel shows the experimental *SBR* estimated for the emitter after spectral filtering. (c) Temporal evolution of the PL signal of the emitter over 5 minutes. (d) Temporal evolution of the integrated PL intensity of the emitter after spectral filtering over 5 minutes. The dashed line and the yellow shadowed area represent the average value and the standard deviation of the integrated PL intensity, respectively.
